# Fifteen years of autoimmune encephalitis in Denmark: incidence, epidemiology, and trends in treatment

**DOI:** 10.1007/s00415-026-13979-8

**Published:** 2026-07-23

**Authors:** Thomas Agerbo Gaist, Mette Scheller Nissen, Silke Simone Aalborg Funch, Mette Blok-Andersen, Andrea Gestsdóttir, Christian Jonasson Winterberg, Anna Christine Nilsson, Charlotte Aaberg Poulsen, Morten Blaabjerg

**Affiliations:** 1 Neurology Research Unit, Department of Clinical Research, University of Southern Denmark, Odense, Denmark; 2 Department of Neurology, Odense University Hospital, Odense, Denmark; 3 OPEN – Odense Patient Data Explorative Network, Department of Clinical Research, University of Southern Denmark, Odense, Denmark; 4 Department of Clinical Immunology, Odense University Hospital, Odense, Denmark; 5 Department of Nuclear Medicine, Odense University Hospital, Odense, Denmark

**Keywords:** Autoimmune encephalitis, Neuroinflammation, LGI1, NMDAR, Epidemiology, Incidence

## Abstract

**Introduction:**

Despite increasing awareness of autoimmune encephalitis (AE), real-world, population-based epidemiological data are scarce.

Using 15 years of nationwide, centralized testing data, we aimed to (1) characterize all Danish patients with neuronal cell-surface antibodies (NSAbs), GAD65, and GFAP; (2) estimate AE incidence in Denmark; and (3) assess temporal trends in treatment strategies.

**Methods:**

Nationwide testing data from 2009 to 2023 were obtained from the Danish centralized antibody test center, and the medical records for antibody-positive patients were reviewed. National and regional incidence rates were calculated using census data, and trends in testing, incidence, and treatment were analyzed.

**Results:**

A total of 198 patients were included. The most prevalent antibody was NMDAR (*n* = 79), followed by LGI1 (*n* = 37), GAD65 (*n* = 20), and CASPR2 (*n* = 19).

The national average crude incidence rate (IR) in 2019–2023 was 2.8 cases per million person-years. Incidence increased by 10% annually during the study period (IRR 1.10, 95% CI 1.04–1.17, *P* = 0.001). The highest regional IR was observed in the region hosting the national testing center.

Second-line treatment in NMDARE increased from 24% in 2009–2013 to 48% in 2019–2023. In contrast, use of steroid-sparing therapy declined from 41 to 26% in NMDARE and from 39 to 0% in LGI1.

**Discussion:**

In this nationwide cohort, Danish AE patients were comparable to previously reported cohorts and incidence estimates were similar to those reported in other national studies. Higher incidence in the testing region suggests that local expertise improves case ascertainment. Treatment patterns evolved over time, likely reflecting increasing clinical experience with AE.

**Supplementary Information:**

The online version contains supplementary material available at 10.1007/s00415-026-13979-8.

## Background

Autoimmune encephalitis (AE) comprises a heterogeneous group of inflammatory disorders of the central nervous system characterized by subacute onset of neuropsychiatric symptoms, seizures, cognitive impairment, and movement disorders. Over the past two decades, the identification of autoantibodies targeting neuronal surface and synaptic antigens has fundamentally transformed the understanding, diagnosis and treatment of encephalitis previously classified as idiopathic.

Despite increasing awareness, AE remains diagnostically challenging due to its broad clinical spectrum. Consensus diagnostic criteria have improved clinical recognition, but access to comprehensive antibody diagnostics and variation in testing strategies across centers continue to influence case ascertainment and reported incidence [15, 24].

Epidemiological studies from Europe and North America suggest that the incidence of AE is comparable to that of infectious encephalitis and may even exceed it in certain age groups [9]. Importantly, several studies have demonstrated a temporal increase in the number of diagnosed cases, which is thought to reflect improved diagnostic capabilities, broader antibody panels and heightened clinical awareness rather than a true rise in disease occurrence. However, many existing cohorts are limited by regional recruitment, referral bias, or incomplete diagnostic coverage, complicating longitudinal assessment of diagnostic trends and treatment practices.

Parallel to advances in diagnostics, the therapeutic landscape of AE has evolved substantially. Early initiation of immunotherapy including high-dose corticosteroids, intravenous immunoglobulin and plasma exchange has been associated with improved outcomes, while second-line agents such as rituximab and cyclophosphamide are increasingly used in refractory disease [29]. Nonetheless, evidence guiding treatment strategies remains largely observational, and real-world data on changes in therapeutic practice over time are scarce.

National, population-based cohorts with complete diagnostic capture are therefore essential to accurately describe the epidemiology, diagnostic activity and treatment evolution of AE. The Danish Autoimmune Encephalitis (DANAE) registry is a national cohort and includes sero-positive AE cases from 2009 and onward. The DANAE registry represents a unique and largely unbiased dataset, as all antibody testing is performed through a single national reference laboratory. All cases are assessed clinically to ensure that clinical features are compatible with antibody results. This structure ensures comprehensive inclusion of all diagnosed patients within the country and allows precise quantification of temporal trends in diagnostic testing volume, confirmed AE cases, treatment strategies, and the distribution of specific AE subtypes. Such a design minimizes referral and selection bias and provides a robust framework for evaluating how advances in immunological diagnostics have translated into clinical practice at a population level.

## Methods

### Study setting

Denmark has 6.024.684 inhabitants (Statistics Denmark, November 2025), provides universal, free healthcare and has nationwide, centralized AE-related antibody testing. Thus, Denmark provides an excellent setting for studying the incidence of AE.

### Antibody testing

Patients with neuronal surface antibodies (NSAbs), GFAP and GAD65, were included in the study.

All autoantibodies were detected using at least two test modalities. NSAbs were tested with commercial cell-based assays (CBA) (Euroimmun, Lübeck, Germany) and confirmed with commercial tissue-based assays (TBA) (Euroimmun). From 2019 and onward, samples with IIF on TBA compatible with GFAP autoantibodies were sent to Euroimmun for confirmatory CBA testing. GAD65 autoantibodies were tested with TBA, CBA and/or line immunoassay (LIA). Early in the study period, GAD65 was tested using radioimmunoassay (RIA) (cutoff > 2000 IU/mL).

Samples with equivocal indirect immunofluorescence (IIF) patterns on CBA or TBA were sent to Euroimmun for further testing and possible confirmatory testing using specific CBAs.

Data on all positive antibody tests were available for the whole study period. Data on the number of individuals tested and whether they were tested in serum, CSF, or both were available from 2012 to 2023, while the number of tests conducted (i.e., all tests performed for each individual) was only available from 2014 to 2023.

### Patient inclusion

Data on positive CSF and serum samples from 2009 to 2023 were extracted from national testing data. Patients with corresponding phenotypes were then identified using medical records and detailed data on symptoms, work-up, treatment and outcome included in the database. Patients were included if they fulfilled the 2016 Graus criteria for definite AE[15]. Both encephalitic and non-encephalitic phenotypes were eligible for inclusion and an expert (MBL) reviewed patients with non-encephalitic, uncommon, or ambiguous phenotypes before inclusion.

We collected individual-level information on demographics, clinical presentation, paraclinical testing and treatment. Functional status was assessed at peak disease using the modified Rankin Scale (mRS) and the Clinical Assessment Scale for autoimmune encephalitis (CASE). Additionally, we estimated a premorbid mRS and an mRS at latest follow-up based on information from medical charts.

For registration of treatment we considered high-dose IV steroids, plasmapheresis (PLEX), and IV immunoglobulin therapy (IVIG) either alone or in combination as first-line treatment. Second-line treatment included rituximab and/or cyclophosphamide. Steroid-sparing agents included azathioprine, methotrexate, and mycophenolate mofetil.

Patients were grouped by antibodies. Patients double-positive for anti-LGI1 and anti-CASPR2 were treated as a separate group.

### Statistical analysis

Data were collected using REDCap and statistical analysis was performed in STATA BE 19.5 and R (version 4.5.1) on a secure server provided by Open Patient Explorative Network (OPEN). Choropleth maps were generated using the ggplot2 and sf packages in R.

Demographics were presented using median and interquartile range (IQR) for numerical data and percentages for categorical data, unless otherwise specified.

Yearly, overall, crude incidence rates (IR) and 5-year IRs were calculated using census data from Statistics Denmark. Crude IRs were calculated by dividing the number of cases by the total population and exact 95% confidence intervals were calculated assuming a Poisson distribution.

Age- and sex-adjusted IRs were calculated using direct standardization with the average Danish population (2009–2023) stratified by 5-year age bands and sex.

Poisson regression models were initially specified to examine temporal trends in AE case counts and in the total number of individuals tested with calendar year as the independent variable and the natural logarithm of the background population size as offset.

Because of substantial overdispersion in both models, negative binomial regressions with the same covariate structure were fitted instead. Results were reported as incidence rate ratios (IRR) with 95% confidence intervals.

To examine the change in proportion of patients tested in serum and CSF over time, a generalized linear model with a binomial distribution and logistic link function was fitted, with the total individuals tested as offset.

To describe treatment patterns over time, annual proportions of patients receiving first-line, second-line and steroid-sparing maintenance treatment were summarized descriptively.

## Results

**Fig. 1:**
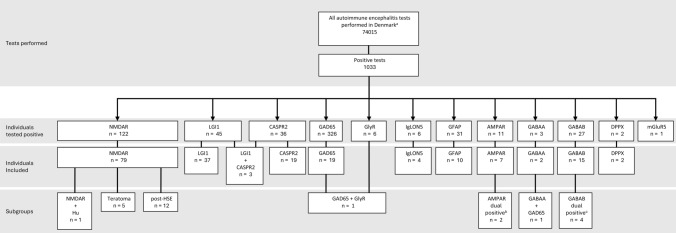
Flowchart **a** Tests performed in Denmark from 2014 to 2023 **b** AMPAR + NMDAR (*n* = 1), AMPAR + GABAB (*n* = 1) **c** GABAB + Hu (*n* = 1), GABAB + SOX1 (*n* = 2), GABAB + SOX1 + ZIC4 (*n* = 1) NMDAR: N-methyl-D-aspartate receptor; LGI1: Leucine-rich glioma-inactivated 1; GAD65: Glutamic acid decarboxylase 65; GlyR: Glycine receptor; CASPR2: Contactin-associated protein 2; GABAB: Gamma-aminobutyric acid type B receptor; GFAP: Glial fibrillary acidic protein; AMPAR: α-amino-3-hydroxy-5-methyl-4-isoxazolepropionic acid receptor; IgLON5: Immunoglobulin-like cell adhesion molecule 5; GABAA: Gamma-aminobutyric acid type A receptor; DPPX: Dipeptidyl-peptidase-like protein 6; SOX1: Sry-like high-mobility group box 1; Zic4: Zinc finger protein ZIC 4

**Fig. 2:**
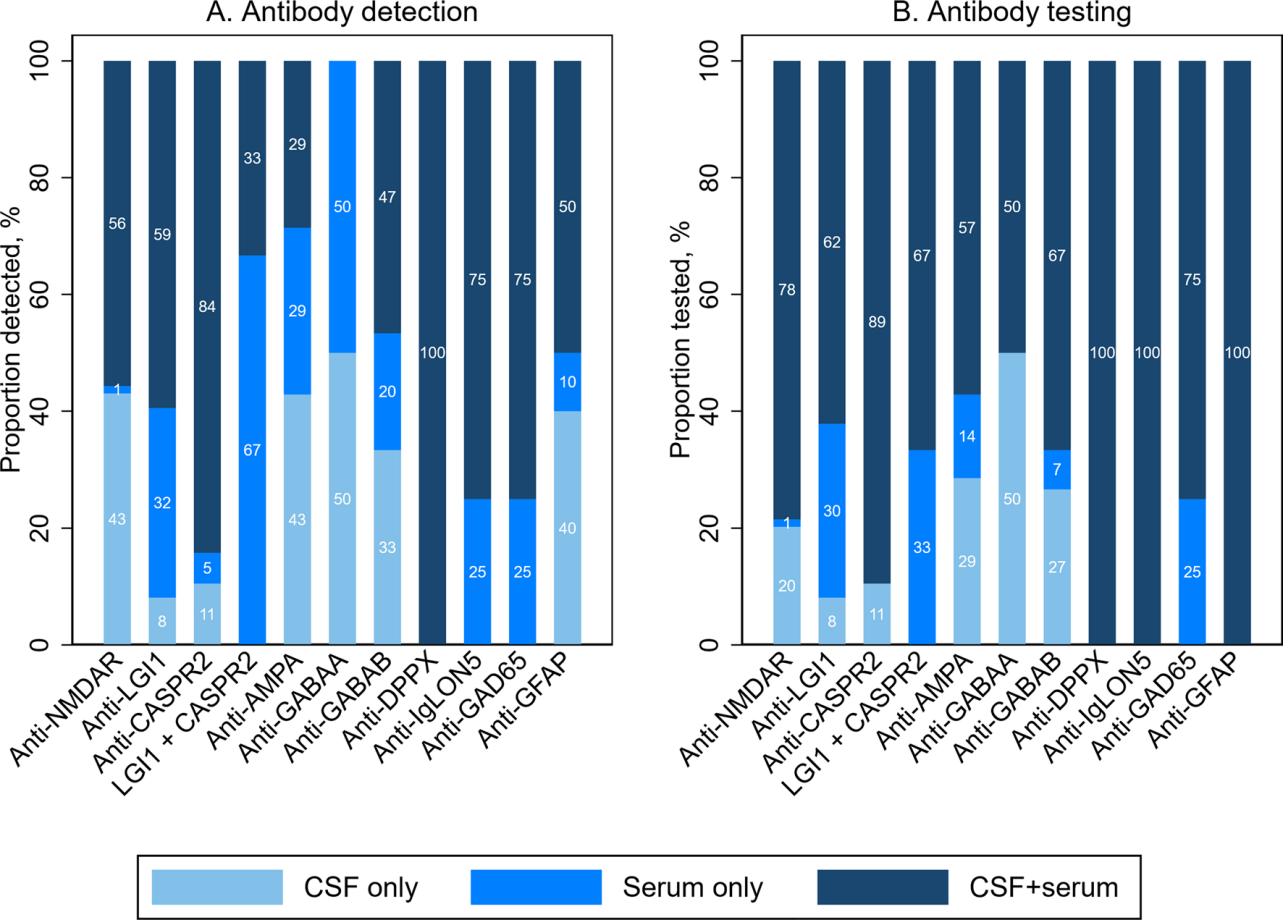
Antibody detection and testing among included patients Proportion of included patients in which antibodies were (**A**) detected or (**B**) tested for in serum only, CSF only or both

In all, 74,015 antibody tests were performed on 21.714 samples. During the study period, 1033 tests were positive for AE-related antibodies in 604 individuals in Denmark. After review of the medical records, 198 patients were included (Fig. 1). None of the excluded patients fulfilled the clinical criteria for AE and many false positives were attributable to patients referred for AE testing by mistake as part of an endocrinological evaluation for the presence of diabetes-related GAD65 in serum. For an overview of the antibody distribution among excluded patients, see eFig. 1. Clinical characteristics, paraclinical testing and outcomes are presented in Table 1, Table 2, and Table 3, respectively. For a visual overview of the detection and testing rates in CSF and serum among included patients, see Fig. 2.

**Table 1:** Clinical characteristics

	NMDAR	LGI1	CASPR2	LGI1 + CASPR2	GAD65	GABAB	GFAP	AMPAR	IgLON5	GABAA	DPPX
Number (*n*)	79	37	19	3	20	15	10	7	4	2	2
Age, median(range)	29 (3–81)	67 (30–84)	70 (4–78)	50 (47–66)	45 (9–69)	71 (54–88)	58 (24–74)	61 (25–84)	74 (61–81)	48 (43–53)	55 (49–61)
Sex, % female (female/male)	57 (45/34)	41 (15/22)	5 (1/18)	33 (1/2)	50 (10/10)	40 (6/9)	30 (3/7)	43 (3/4)	25 (1/3)	50 (1/1)	0 (0/2)
Phenotypes	NMDARE: 78 Meningoencephalitis: 1	LE: 37	Encephalitis: 1 Isaac's: 1 LE: 15 Morvan: 1 PNP: 1	Isaac's: 2 LE: 1	Cerebellar degeneration: 4 Encephalitis unspecified: 2 LE: 6 SPSD 8	LE: 15	Encephalomyelitis: 5 LE: 1 Meningoencephalitis: 2 Myelopathy: 1 OMS: 1	LE: 7	Chorea with dementia: 1 Bulbar syndrome and or external opthalmoplegia: 2 Motor neuron syndrome: 1	Cortical/subcortical encephalitis: 1 LE: 1	PERM: 2
Cognitive symptoms, %	97 (77/79)	100 (37/37)	89 (17/19)	33 (1/3)	28 (5/18)	93 (14/15)	90 (9/10)	100 (7/7)	75 (3/4)	50 (1/2)	100 (2/2)
Psychiatric symptoms, %	81 (63/78)	66 (23/35)	58 (11/19)	33 (1/3)	47 (9/19)	67 (10/15)	44 (4/9)	43 (3/7)	25 (1/4)	0 (0/2)	50 (1/2)
Movement disorder, %	53 (41/78)	49 (18/37)	37 (7/19)	33 (1/3)	5 (1/20)	20 (3/15)	40 (4/10)	14 (1/7)	50 (2/4)	0 (0/2)	100 (2/2)
Language disorder, %	65 (50/77)	22 (8/36)	11 (2/19)	0 (0/3)	20 (4/20)	47 (7/15)	30 (3/10)	29 (2/7)	75 (3/4)	0 (0/2)	50 (1/2)
sleep disorder, %	39 (29/75)	20 (6/30)	41 (7/17)	0 (0/3)	11 (2/18)	15 (2/13)	22 (2/9)	29 (2/7)	75 (3/4)	0 (0/2)	0 (0/2)
Seizures, %	63 (47/75)	92 (33/36)	74 (14/19)	33 (1/3)	37 (7/19)	87 (13/15)	22 (2/9)	57 (4/7)	25 (1/4)	50 (1/2)	0 (0/2)
Autonomic symptoms, %	42 (32/77)	44 (15/34)	50 (9/18)	0 (0/3)	28 (5/18)	27 (4/15)	44 (4/9)	43 (3/7)	25 (1/4)	0 (0/2)	0 (0/2)
Peripheral symptoms, %	10 (8/79)	16 (6/37)	58 (11/19)	67 (2/3)	15 (3/20)	0 (0/15)	20 (2/10)	0 (0/7)	25 (1/4)	0 (0/2)	50 (1/2)
ICU admission, %	28 (22/79)	11 (4/37)	11 (2/19)	0 (0/3)	0 (0/20)	47 (7/15)	30 (3/10)	43 (3/7)	50 (2/4)	0 (0/2)	0 (0/2)
Time to treatment, days, median(IQR)	23 (11–46)	104 (51–197)	193 (40–367)	26 (26–26)	328 (128–2879)	16 (14–36)	20 (9–132)	18 (16–21)	3035 (315–4393)	12 (11–14)	26 (16–35)

*NMDAR* N-methyl-D-aspartate receptor, *NMDARE* N-methyl-D-aspartate receptor encephalitis, *LGI1* Leucine-rich glioma-inactivated 1, *GAD65* Glutamic acid decarboxylase 65, *CASPR2* Contactin-associated protein 2, *GABAB* Gamma-aminobutyric acid type B receptor, *GFAP* Glial fibrillary acidic protein, *AMPAR* α-amino-3-hydroxy-5-methyl-4-isoxazolepropionic acid receptor, *IgLON5* Immunoglobulin-like cell adhesion molecule 5, *GABAA* Gamma-aminobutyric acid type A receptor, *DPPX* Dipeptidyl-peptidase-like protein 6, *LE* Limbic encephalitis, *OMS* Opsoclonus-myoclonus syndrome, *PERM* Progressive encephalomyelitis with myoclonus and rigidity, *PNP* Peripheral polyneuropathy, *SPSD* Stiff-person syndrome disorder

**Table 2:** Paraclinical testing

	NMDAR	LGI1	CASPR2	LGI1 + CASPR2	GAD65	GABAB	GFAP	AMPAR	IgLON5	GABAA	DPPX
Number(*n*)	79	37	19	3	20	15	10	7	4	2	2
Brain MRI*	43 (34/79)	62 (23/37)	44 (8/18)	100 (1/1)	39 (7/18)	60 (9/15)	67 (6/9)	33 (2/6)	50 (2/4)	100 (2/2)	0 (0/2)
Brain FDG-PET*	87 (20/24)	94 (16/17)	100 (6/6)	100 (1/1)	100 (3/3)	100 (4/4)	50 (1/2)	75 (3/4)	100 (1/1)	100 (1/1)	. (0/0)
Whole-body FDG-PET*	22 (8/36)	29 (8/28)	39 (7/18)	100 (2/2)	9 (1/11)	100 (10/10)	62 (5/8)	20 (1/5)	0 (0/1)	0 (0/1)	100 (1/1)
EEG*	91 (64/70)	86 (31/36)	80 (12/15)	100 (1/1)	88 (7/8)	93 (14/15)	100 (4/4)	43 (3/7)	100 (1/1)	100 (2/2)	0 (0/2)
CSF pleocytosis*	87 (69/79)	14 (5/37)	37 (7/19)	0 (0/2)	50 (8/16)	60 (9/15)	90 (9/10)	57 (4/7)	25 (1/4)	0 (0/2)	50 (1/2)
CSF cell count**	47 (7–436)	12 (6–44)	8 (6–30)		8 (6–14)	36 (6–148)	120 (10–804)	30 (12–66)	7 (.)		6 (.)
Oligoclonal bands*	67 (18/29)	30 (3/10)	17 (1/6)	. (0/0)	88 (7/8)	100 (4/4)	83 (5/6)	100 (2/2)	50 (1/2)	. (0/0)	100 (1/1)

^*^ %abnormal (abnormal/total)

^**^ median(min–max)

*NMDAR* N-methyl-D-aspartate receptor, *LGI1* Leucine-rich glioma-inactivated 1, *GAD65* Glutamic acid decarboxylase 65, *CASPR2* Contactin-associated protein 2, *GABAB* Gamma-aminobutyric acid type B receptor, *GFAP* Glial fibrillary acidic protein, *AMPAR* α-amino-3-hydroxy-5-methyl-4-isoxazolepropionic acid receptor, *IgLON5* Immunoglobulin-like cell adhesion molecule 5, *GABAA* Gamma-aminobutyric acid type A receptor, *DPPX* Dipeptidyl-peptidase-like protein 6

**Table 3:** Outcomes in antibody-mediated disease in Denmark

	NMDAR	LGI1	CASPR2	LGI1 + CASPR2	GAD65	GABAB	GFAP	AMPAR	IgLON5	GABAA	DPPX
Number(n)	79	37	19	3	20	15	11	7	4	2	2
Follow-up time, months*	38 (18–73)	73 (26–132)	35 (17–66)	56 (52–60)	90 (42–126)	3 (1–13)	16 (10–35)	58 (30–84)	136 (88–170)	48 (44–52)	14 (12–17)
Premorbid emRs*	0 (0–2)	0 (0–0)	0 (0–0)	0 (0–0)	0 (0–0)	0 (0–2)	0 (0–0)	0 (0–0)	2 (2–2)	2 (1–2)	1 (0–2)
Peak disease emRS*	4 (4–5)	3 (3–3)	3 (2–3)	2 (2–2)	3 (2–3)	5 (4–5)	4 (3–5)	5 (4–5)	4 (3–5)	2 (2–3)	4 (4–4)
Peak disease CASE score*	7 (6–11)	5 (4–6)	5 (4–5)	0 (0–2)	4 (3–5)	9 (6–14)	6 (2–10)	9 (4–10)	6 (5–7)	3 (2–4)	8 (7–10)
Post disease rmRS*	1 (0–2)	2 (1–3)	2 (1–3)	0 (0–0)	2 (2–3)	6 (6–6)	2 (1–4)	2 (2–6)	6 (5–6)	2 (2–3)	3 (3–3)
Relapse rate, %	10 (8/79)	22 (8/37)	37 (7/19)	0 (0/3)	15 (3/20)	7 (1/15)	18 (2/11)	29 (2/7)	0 (0/4)	0 (0/2)	0 (0/2)
Malignancy/tumor, %	11 (9/79)	11 (4/37)	21 (4/19)	33 (1/3)	0 (0/20)	93 (14/15)	18 (2/11)	57 (4/7)	0 (0/4)	50 (1/2)	50 (1/2)
1-year mortality**, %	9 (6/68)	3 (1/34)	18 (3/17)	0 (0/2)	0 (0/12)	62 (8/13)	25 (2/8)	0 (0/6)	50 (2/4)	0 (0/2)	0 (0/1)

^*^ Median (IQR)

^**^ Death from autoimmune encephalitis or associated cancer

*NMDAR* N-methyl-D-aspartate receptor, *LGI1* Leucine-rich glioma-inactivated 1, *GAD65* Glutamic acid decarboxylase 65, *CASPR2* Contactin-associated protein 2, *GABAB* Gamma-aminobutyric acid type B receptor, *GFAP* Glial fibrillary acidic protein, *AMPAR* α-amino-3-hydroxy-5-methyl-4-isoxazolepropionic acid receptor, *IgLON5* Immunoglobulin-like cell adhesion molecule 5, *GABAA* Gamma-aminobutyric acid type A receptor, *DPPX* Dipeptidyl-peptidase-like protein 6, *CASE* Clinical Assessment Scale in Autoimmune Encephalitis, *emRS* estimated modified Rankin Scale

### Anti-N-methyl-D-aspartate receptor encephalitis (NMDARE)

The median age at disease onset was 29 (18–56) years and 57% (45/79) were female.

NMDARE patients (*n* = 79) had a median peak mRS of 4 (4–5) and CASE score of 7 (6–11). Intensive care unit (ICU) admission was required in 28% (22/79) of patients, with more than half requiring mechanical ventilation (55% 12/22). The median treatment delay was 23 (11–46) days.

Patients experienced a prodromal phase in 27% (21/78) of cases with headache, gastrointestinal (GI) symptoms, and fever being the most common symptoms.

An underlying trigger was identified in 28% (22/79). Sixteen percent (13/79) of patients had antecedent viral encephalitis, including herpes simplex virus type 1 encephalitis (HSE) (*n* = 12) and tick-borne encephalitis (TBE) (*n* = 1) [12]. An underlying neoplasm was identified in 11% (9/79). Of these, ovarian teratomas were found in 11% (5/45) of female cases. Other neoplasms identified included small-cell lung cancer (SCLC) (*n* = 1), diffuse large B-cell lymphoma + follicular thyroid carcinoma (*n* = 1), disseminated prostate cancer (*n* = 1), and breast cancer (*n* = 1). There was no overlap between patients with antecedent viral encephalitis and underlying tumors.

A majority of NMDARE patients had monocytic pleocytosis (> 5 × 10^6^ cells/L) in CSF (87% 69/79) with a median leukocyte count of 47 (range 7–436) × 10^6^ cells/L in those with pleocytosis. Further, oligoclonal bands (OCB) were present in CSF in 67% (18/27) of those tested.

Inflammatory MRI changes, defined as T2/FLAIR hyperintensities, contrast enhancement, or swelling, were found in 33% (22/66) of those without prior infectious encephalitis. Brain FDG-PET was abnormal in 75% (18/24) most frequently showing changes in the occipital region (67% 12/18), followed by the temporal (44% 8/18), frontal (33% 6/18) and parietal (33% 6/18) regions.

In patients with no inflammatory changes on MRI, brain PET identified abnormal metabolism in 77% (10/13).

EEGs were abnormal in 91% (64/70) of patients. The most common finding was non-specific focal/diffuse slow wave activity (91% 58/64). Other findings included periodic epileptiform discharges (20% 13/64) and convulsive/non-convulsive status epilepticus (12% 8/64). Extreme delta brush, a distinctive EEG pattern specific to NMDARE, was described in 6% (4/64).

The 1-year mortality rate, defined as deaths related to the disease or an underlying malignancy, was 9% (6/69). The relapse rate was 10% (8/79) and follow-up, median mRS was 1 (0–2).

### Anti-leucine-rich glioma-inactivated 1 (LGI1)

LGI1 patients (*n* = 37) were older (67 (60–74)) with a higher proportion of male patients (41% female 15/37), compared to NMDARE. Peak disease functional scores were lower (mRS 3 (3–3), CASE 5 (4–6)) and only 12% (4/33) were admitted to the ICU. Time-to-treatment was considerably longer with a median delay of 104 (51–196) days.

All patients had cognitive dysfunction with memory deficits (95% 35/37) being the most prominent feature. FBDS were noted in 38% (14/37) and other movement disorders in 14% (5/37). Most patients (83% 30/36) developed hyponatremia at some point in the acute phase.

An underlying malignancy was found in 11% (4/37). Malignancies included breast cancer (*n* = 1), prostate cancer (*n* = 1), polycythemia vera (*n* = 1) and a pancreatic neuroendocrine tumor (*n* = 1).

CSF pleocytosis was only present in 14% (5/37) of patients with low CSF leukocyte levels (12 (range 6–44) × 10^6^ cells/L) in those with pleocytosis. OCBs were present in a third of tested patients (30% 3/10).

Brain MRI showed signs of neuroinflammation in 62% (23/37) of patients. The most common finding was temporal lobe changes (96% 22/23). Brain FDG-PET revealed abnormal brain metabolism in 94% (16/17) of tested patients. Similarly, changes in the temporal lobe were most frequently observed (94% 15/16), followed by changes in the basal ganglia (19% 3/16).

In this group, three patients with no neuroinflammation on MRI had metabolic changes on PET imaging.

EEGs were abnormal in 86% (31/36) of patients with non-specific low-frequency findings being the most frequent finding (90% 28/31). Periodic epileptiform discharges were present in more than half of abnormal EEGs (58% 18/31) while status epilepticus was found in only 10% (3/31).

Median follow-up mRS was 2 (1–3), relapse rate was 22% (8/37) and 1-year mortality was 3% (1/34).

### Anti-contactin-associated protein 2 (CASPR2)

Patients with CASPR2-mediated neurological disease (*n* = 19) had a median age of 70 (63–76) and were almost exclusively male (5% female, 1/19).

Peak disease severity was similar to that of LGI1 patients with a median mRS of 3 (2–3) and CASE score of 5 (4–5). Median time from symptom onset to treatment was 193 (82–360) days.

A majority of patients presented with cognitive symptoms (89% 17/19) and seizures (74% 14/19) and approximately half of patients presented with peripheral neuropathy and/or neuromyotonia (58% 11/19). Other frequent manifestations were psychiatric symptoms (58% 11/19), autonomic dysfunction (50% 9/18) and movement disorders (32% 6/19).

The rate of underlying cancer was 21% (4/19) and included thymoma and prostate cancer.

CSF had low-level pleocytosis in 39% (7/18) with a median leukocyte count of 8 (range 6–30) × 10^6^ cells/L.

Neuroinflammatory changes were found in 44% (8/18) of brain MRIs, while all of the brain FDG-PET scans performed (6/6) revealed brain metabolic changes compatible with AE, mostly in the temporal region (83% 5/6).

Most of the EEGs performed were considered abnormal (80% 12/15) with low frequency patterns (75% 9/12), epileptiform discharges (58% 7/12), and status epilepticus (16% 2/12).

CASPR2 patients had a median follow-up mRS of 2 (1–3) and 1-year mortality of 18% (3/17). Patients in this group had the highest relapse rate in the cohort (37% 7/19).

Patients positive for both CASPR2 + LGI1 (*n* = 3) were analyzed separately and are presented in Table 1, Table 2, and Table 3.

### Anti-glutamic acid decarboxylase 65 (GAD65)

GAD65-related neurological disease (*n* = 20) was phenotypically heterogeneous. The median age was 45 (28–52) and 50% were female. Time to treatment was very long with a median of 328 (141–2264) days.

The most prominent phenotype was stiff-person syndrome disorder (SPSD), seen in 8 patients, followed by encephalitis (*n* = 8) and cerebellar degeneration (*n* = 4). First-line treatment was administered to all encephalitic patients (8/8) and 75% of both SPSD (6/8) and cerebellar ataxia (3/4). Second-line therapy was given in 38% (3/8) of encephalitis patients, whereas 25% (2/8) SPSD patients and no cerebellar ataxia patients received second-line treatment. Most patients with encephalitic phenotypes obtained mRS < 3 (88% 7/8), while this was true in 63% (5/8) of SPSD and 25% (1/4) of patients with cerebellar ataxia.

None of the patients had underlying malignancies and there were no deaths within the first year of follow-up.

### Anti-aminobutyric acid type B receptor (GABAB)

Patients with GABAB antibodies (*n* = 15) had a median age of 71 (58–77) years and male predominance (40% female).

This patient group was characterized by severe peak disease, median mRS 5 (3–5) and CASE 9 (6–15) with more than half of patients requiring ICU treatment (54% 7/13). Treatment delay was shorter compared to LGI1 and CASPR2 with 16.5 (14–36) days.

Patients had limbic encephalitis phenotype that manifested primarily with cognitive dysfunction (93% 14/15) and seizures (87% 13/15), with a fifth of patients having confirmed status epilepticus on EEG (20% 3/15).

GABAB had the highest rate of underlying malignancy in the cohort (93% 14/15). The following cancers were identified in the group: SCLC (*n* = 11), unspecified lung cancer (*n* = 1), tongue root cancer (*n* = 1), disseminated prostate cancer (*n* = 1).

Moreover, patients in this group had the highest overall one-year mortality (62% 8/13). Relapse rate was 7% (1/15).

### Anti-glial fibrillary acidic protein (GFAP)

Patients with GFAP (*n* = 10) had a median age of 58 (38–71) and were primarily male (30% female 3/10). Patients predominantly presented with acute onset of encephalitis with combinations of meningitis or myelopathy with median mRS 4 (3–5) and CASE score 6 (2–10). A third of patients (30% 3/10) were admitted to the ICU with all of these patients needing mechanical ventilation.

Patients had an inflammatory CSF profile with 82% (9/11) pleocytosis, high monocytic leukocyte counts, median 120 (range 10–804), and oligoclonal bands in 83% (5/6) of patients tested. Of those tested in the initial phase of the disease, CSF lactate was elevated in 80% (4/5) while no patients had hypoglychorrachia (0/6).

Most patients presented with a prodromal phase 70% (7/10) with fever and headache (72% 5/7) and GI symptoms (43% 3/7).

Tumors were found in 18% (2/11): prostate cancer (*n* = 1), prostate cancer + disseminated malignant melanoma (*n* = 1). Relapses occurred in 18% (2/11) and 1-year mortality was 25% (2/8).

### Anti-α-amino-3-hydroxy-5-methyl-4-isoxazolepropionic acid receptor (AMPAR)

The median age of AMPAR encephalitis patients (*n* = 7) was 61 (47–76) years and 43% (3/7) of patients were female. Peak disease severity was similar to GABAB with a median mRS of 5 (3–5) and CASE 9 (4–10) and 43% (3/7) ICU admission rate.

Clinically, patients presented with a limbic encephalitis with prominent short-term memory deficits (100% 7/7). Seizures were seen in 57% (4/7) of patients and psychiatric and autonomic symptoms were described in 43% (3/7) of patients.

An underlying neoplasm was identified in 57% (4/7): SCLC (*n* = 1), unspecified lung cancer (*n* = 2), thymoma (*n* = 1).

There were no deaths related to AE or an underlying cancer within the first year of follow-up and 29% (2/7) relapsed.

Baseline characteristics, paraclinical testing and outcomes for IgLON5 (*n* = 4), GABAA (*n* = 2), and DPPX (*n* = 2) are reported in Tables 1, 2, and 3, respectively.

### Epidemiology

There was a 10% increase in crude IR per year (IRR = 1.10, 95% CI 1.04; 1.17, *P* = 0.001) from 2009 to 2023. In 2009 the observed, crude IR was 0.2 (95% CI 0.0—1.0) cases/million person-years, this increased to 5.4 (3.7—7.6) in 2023. Five-year average IR also increased over time from 1.3 (0.9—1.8) cases/million person-years in 2009–2013 to 2.3 (1.8—2.9) in 2014–2018 and 2.8 (2.2—3.5) in 2019–2023.

During 2009–2023, the 5-year average IRs for the most common antibody types were 1.1 (0.7–1.5) for NMDARE, 0.4 (0.2–0.8) for LGI1, 0.3 (0.2–0.6) for CASPR2 and 0.1 (0.0–0.3) for GAD65-mediated encephalitis. There was an increase in the 5-year average IR of patients with SPSD from 0.04 in 2009–2013 to 0.20 in 2019–2023 and the IR for CASPR2 also increased from 0.1 in 2009–2013 to 0.3 in 2019–2023. For an overview of 5-year IRs, see Table 4. Age- and sex-adjusted incidence rates were nearly identical to crude rates, see Table 5.

**Table 4:** Five-year average crude incidence rates of antibody-mediated neurological disease in Denmark

Time interval	Total incidence*	Encephalitis	NMDAR	LGI1	CASPR2	Non-encephalitic phenotypes
2009–2013	1.3 (0.9—1.8)	1.3 (0.9—1.8)	0.6 (0.4—1.0)	0.5 (0.2—0.8)	0.1 (0.0—0.3)	0.0 (0.0—0.2)
2014–2018	2.4 (1.9—3.0)	2.3 (1.8—2.9)	1.1 (0.7—1.5)	0.4 (0.2—0.7)	0.2 (0.1—0.4)	0.1 (0.0—0.3)
2019–2023	3.2 (2.6—3.9)	2.8 (2.2—3.4)	1.1 (0.7—1.5)	0.4 (0.2—0.8)	0.3 (0.2—0.6)	0.4 (0.2—0.7)

^*^ Including encephalitis and non-encephalitic phenotypes, e.g., stiff-person syndrome disorder. Autoantibodies include neuronal cell-surface antibodies, GFAP and GAD65

Incidence rate units are cases/1.000.000 person-years

*NMDAR* N-methyl-D-aspartate receptor, *LGI1* Leucine-rich glioma-inactivated 1, *CASPR2* Contactin-associated protein 2, *GAD65* Glutamic acid decarboxylase 65

**Table 5:** Crude and age- and sex-adjusted incidence rates

	Total incidence*		NMDAR		LGI1	
	Crude	Adjusted	Crude	Adjusted	Crude	Adjusted
2009–2013	1.3 (0.9—1.8)	1.3 (0.9—1.8)	0.6 (0.4—1.0)	0.6 (0.3—0.9)	0.5 (0.2—0.8)	0.5 (0.2—0.7)
2014–2018	2.4 (1.9—3.0)	2.4 (1.8—2.9)	1.1 (0.7—1.5)	1.1 (0.7—1.5)	0.4 (0.2—0.7)	0.4 (0.2—0.7)
2019–2023	3.2 (2.6—3.9)	3.1 (2.5—3.8)	1.1 (0.7—1.5)	1.1 (0.7—1.4)	0.4 (0.2—0.7)	0.4 (0.2—0.6)

^*^ Including encephalitis and non-encephalitic phenotypes, e.g., stiff-person syndrome disorder. Autoantibodies include neuronal cell-surface antibodies, GFAP and GAD65

Incidence rate units are cases/1.000.000 person-years

Incidence rates were adjusted for sex and age in 5-year bands

*NMDAR* N-methyl-D-aspartate receptor, *LGI1* Leucine-rich glioma-inactivated 1

**Fig. 3:**
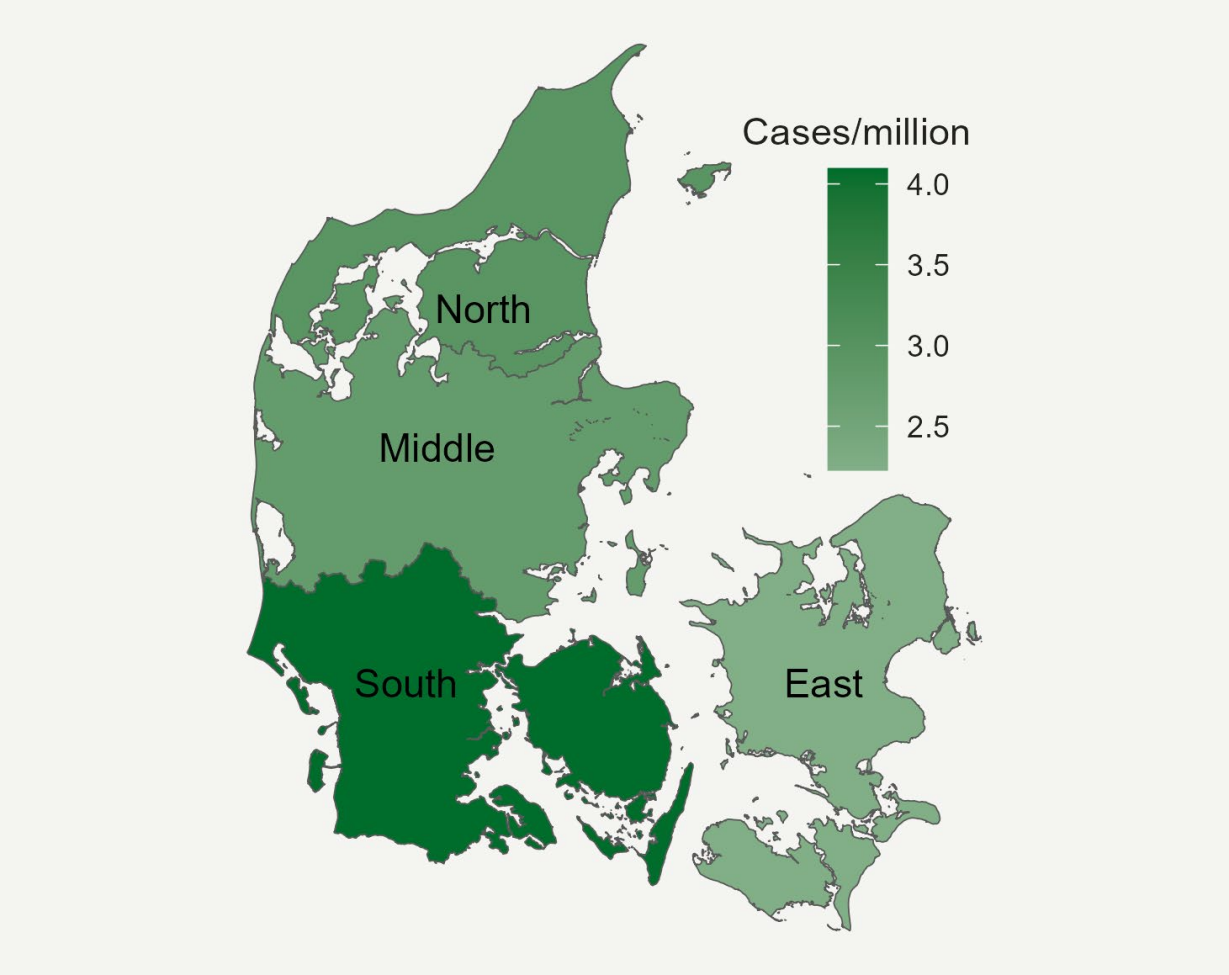
Regional incidence rate of autoimmune encephalitis in Denmark 2019–2023 *North: Region of Northern Denmark; Middle: Region of middle Denmark; South: Region of Southern Denmark; East: Region of Zealand and the Capital region of Copenhagen combined*. Choropleth map of Denmark showing the 5-year average, crude incidence rate (IR) of autoimmune encephalitis in the major regions of Denmark. Patients with neuronal cell-surface autoantibodies, GFAP and GAD65 were included in the study

When examining the age- and sex-adjusted, average 5-year IR by region, we found that the region of Southern Denmark, wherein the national testing is conducted, had the highest IR (3.9) compared to the rest of Denmark, see Fig. 3.

Information on the number of individuals tested was only available from 2012 to 2023. In this period, there was a 23% yearly increase in annual antibody testing in Denmark (IRR = 1.23, 95% CI 1.18; 1.28 *P* < 0.001). Annual testing increased from 189 individuals in 2012 to 2289 individuals in 2023. The proportion of patients tested in both serum and CSF did not increase significantly during the observation period (mean proportion 2012–2023: 32%).

**Fig. 4:**
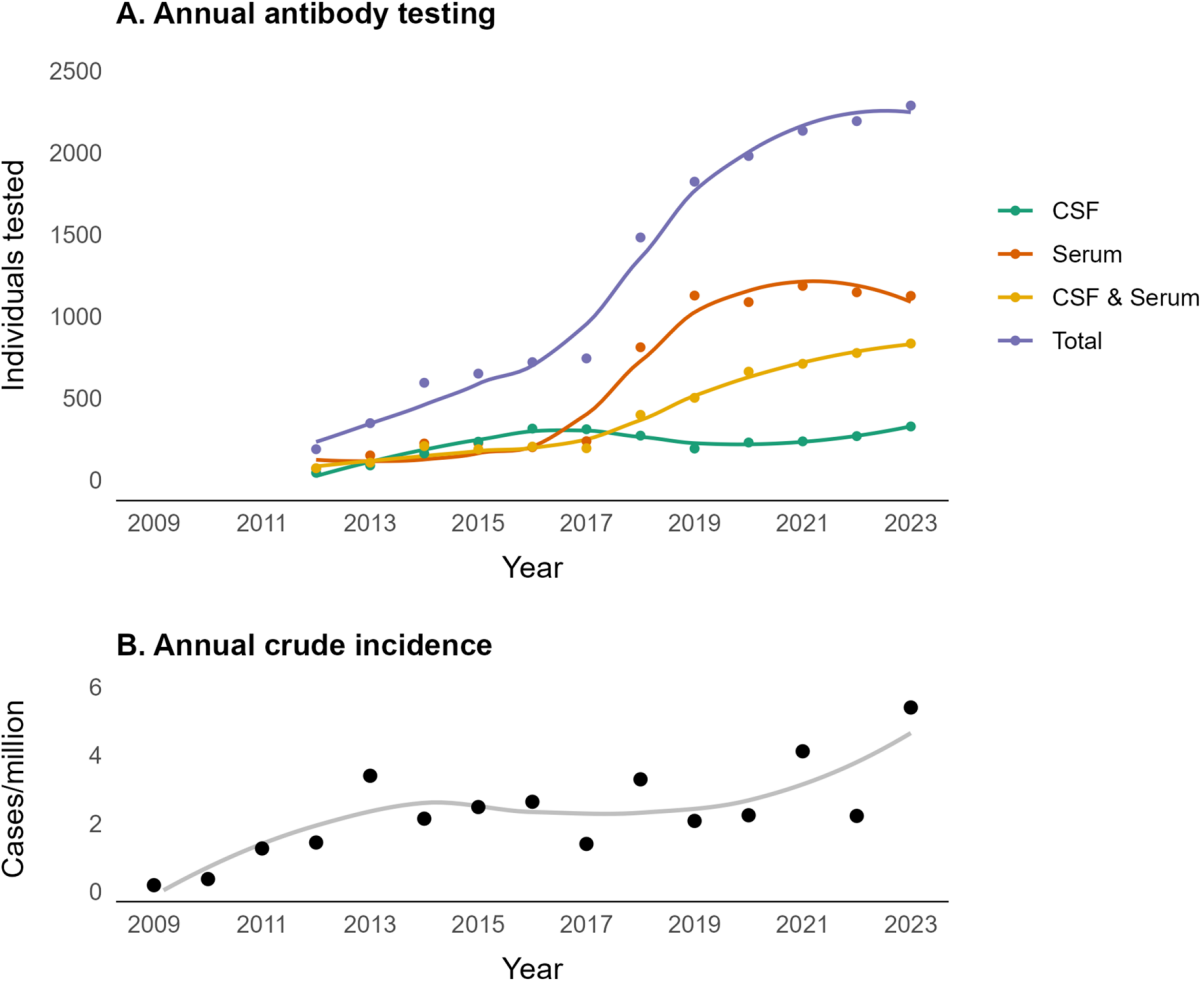
Annual incidence rate of autoimmune encephalitis and individuals tested in Denmark Annual (**A**) number of individuals tested for autoimmune encephalitis (AE)–related antibodies in Denmark and (**B**) crude incidence of AE in cases/million person-years. Patients with non-encephalitic phenotypes, e.g., stiff-person syndrome, are included in this incidence calculation. Testing data were only available from 2012 to 2023. AE-related autoantibodies were defined as neuronal cell-surface antibodies, GFAP and GAD65

For a graphical overview of incidence and AE testing in Denmark, see Fig. 4*.*

### Treatment

Overall, the use of first-line therapy was consistently high among patients with encephalitic phenotypes (92% 169/183) while 24% (44/183) received second-line treatment and a third was treated with steroid-sparing maintenance therapy (33% 60/183).

**Fig. 5:**
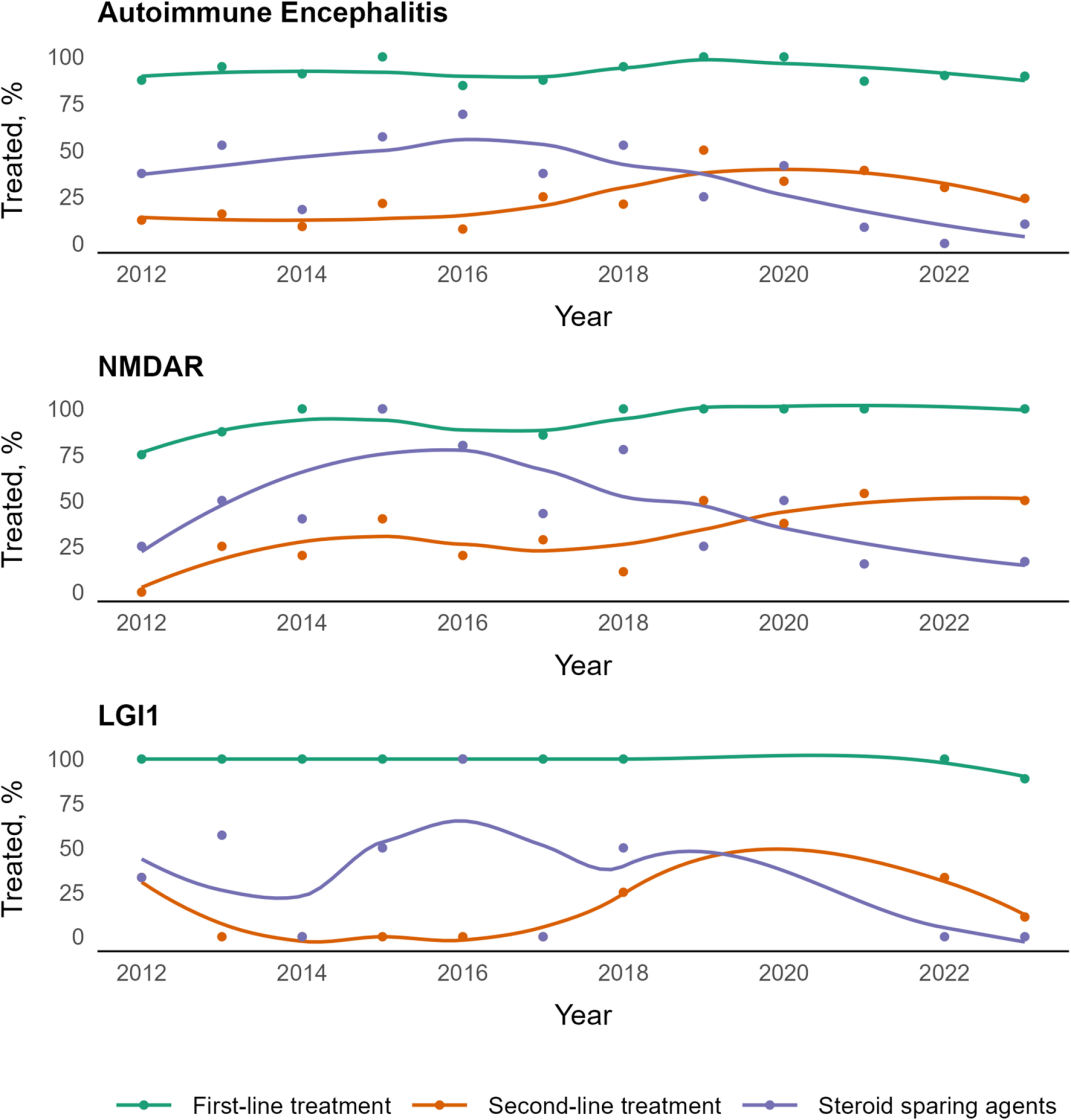
Temporal trends in treatment of patients with autoimmune encephalitis in Denmark First-line treatment included high-dose IV steroids, plasmapheresis (PLEX) and IV immunoglobulin therapy (IVIG). Second-line treatment included rituximab and cyclophosphamide. Steroid-sparing agents included azathioprine, methotrexate and mycophenolate mofetil. AE-related autoantibodies were defined as neuronal cell-surface antibodies, GFAP and GAD65

Notably, even though a third of NMDARE patients received second-line treatment (33% 26/79) a trend toward increasing use of second-line treatment over time appeared with 24% (4/17) of patients receiving second-line treatment in 2009–2013 to 48% (15/31) in 2019–2023, see Fig. 5 for a visual representation.

The use of second-line treatment use in LGI1 was lower at 11% (4/37) with no discernible temporal trends.

The average use of steroid-sparing therapy was high in both groups, 46% (36/79) for NMDARE and 30% (11/37) for LGI1. However, use of steroid-sparing agents seemed to decrease over time in both groups. NMDARE decreased from 41.2% (7/17) in 2009–2013 to 25.8% (8/31) in 2019–2023, with LGI1 decreasing from 38.5% (5/13) to 0% (0/12) in the same period.

## Discussion

In this population-based epidemiological study of AE in Denmark, we characterized all sero-positive patients ever diagnosed in Denmark using nationwide centralized antibody testing data. We demonstrated that (1) the IR of AE in Denmark has increased over time; (2) there was a marked increase in individuals tested annually; (3) the clinical characteristics of Danish AE patients largely align with previously published cohorts; (4) treatment practices of AE patients in Denmark have evolved with increasing use of second-line treatment and decreased use of steroid-sparing agents.

### Cohort characteristics

The NMDARE cohort included in our study largely aligned with previously published cohorts. Compared to a nationwide cohort from the Netherlands, both cohorts had a median age at onset of 29 years, the proportion of post-HSE patients was similar (15% Denmark, 13% the Netherlands), the median treatment delay was identical (3.3 weeks in both cohorts) and the relapse rate was comparable (10% vs 15%). The proportion of women was smaller in our cohort (57% vs. 74%) and the incidence of underlying tumor was lower in Danish patients (11% vs. 25%). Compared to a Chinese cohort published in 2022, there were several similarities: the median age (29 vs. 28 years) and the treatment delay (23 vs. 20 days), the prevalence of underlying tumors (11% vs 12%) and median mRS at peak disease (mRS 4 in both cohorts)[32]. Conversely, there were fewer female patients in the Chinese cohort (57% vs 43%).

The rate of underlying ovarian teratomas among NMDAR patients in Denmark is lower than reported in several previous studies (11% vs ~ 30%[2, 8, 19]). This discrepancy is possibly a result of genetic differences with teratomas being more prevalent among some ethnic groups, such as those of Afro-Caribbean or Asian descent[29]. NMDAR patients are not routinely monitored for the presence of teratomas during follow-up, which could also be a contributing factor to the relatively low prevalence of teratomas.

Among NMDAR patients, 87% (20/24) of brain FDG-PET scans were abnormal. This finding points to PET imaging being superior to brain MRI where abnormalities were found in less than half of patients (43% 34/79). A meta-analysis examining brain PET imaging in AE found similar results with PET abnormalities being present in virtually all NMDAR patients (99%) with MRI changes occurring in approximately a third[23]. Importantly, only a subset of patients received PET imaging in our cohort, possibly inflating the diagnostic yield of brain FDG-PET due to selection bias.

The Danish LGI1 patients resembled other published cohorts [5, 22, 26, 28]. A 2025 paper included two cohorts of LGI1 patients: One from France and a multinational comparison cohort with patients from the Republic of Ireland, the UK, and the USA [5]. The median age of onset (67 years vs. 64–66 years) was comparable to the Danish LGI1 patients and the median mRS was 3 in all three cohorts. The Danish patients had a higher proportion of females (41% vs. 30–31%).

A Spanish cohort of LGI1 patients published in 2025 had similar median age (67 vs. 68 years), proportion of females (41% vs 46%) and peak mRS (both mRS 3) as our LGI1 patients [26]. Other similarities include the proportion of patients with FBDS (39% in our cohort and 43% among Spanish patients) and the proportion of patients with abnormal MRIs (65% vs. 60%).

When comparing our CASPR2 patients with a large international cohort of patients, we again found many similarities [13]: median age (Denmark 70 years vs. study 68 years), proportion of female patients (5% vs. 11%), percentage of patients with pleocytosis (37% vs. 38%), peak disease mRS (both cohorts mRS 3) and the prevalence of movement disorders (32% vs. 36%). CASPR2-mediated disease had the highest relapse rate in the cohort (37% 7/19), which is consistent with previous studies [6, 11]. It may be that the, often insidious, onset of CASPR2-associated disease results in delayed treatment leading to protracted and relapsing disease. It is also possible that CASPR2 by its very nature requires aggressive and prolonged treatment. In fact, a previous study found that CASPR2 relapses were frequent and that long-term maintenance therapy, including multiple courses of rituximab, was often required to prevent relapsing disease [6].

Autoimmune encephalitis mediated by GABA A, GABA B, and AMPAR antibodies can occur in children [16, 17, 25], yet there were no pediatric patients with these antibodies in our cohort. This may be the result of small sample sizes but it may also reflect lack of AE awareness among pediatricians in Denmark.

### Epidemiology

The 5-year IR of AE in Denmark from 2019 to 2023 was 2.8 cases/million person-years. A French epidemiological study from 2020 found that the national IR AE in 2016–2018 was 1.8 [11, 18], while a similar study performed in the Netherlands found that the IR of AE mediated by extracellular antibodies from 2016 to 2021 was 2.96[20]. In the Dutch study, non-encephalitic phenotypes were also included. When including non-encephalitic patients in the Danish IR in 2019–2023, it increases from 2.8 to 3.2. Importantly, VGCC and Tr [DNER] were both included in the extracellular cohort of the Dutch study, while we included GAD65 and GFAP in our AE cohort. Seeing as GAD65-mediated neurological disease is one of the more common antibody-mediated neurological diseases, this could artificially inflate the Danish IR compared to the Dutch. Even if the Dutch study included some antibodies that ours did not. Despite these differences, the overall IRs of AE in Denmark and the Netherlands remain comparable.

In the French study, data from the region of the referral center were analyzed separately because case coverage was the most complete in this region. They found a regional IR of 3.6, indicating that the reported national incidence might underestimate the actual frequency of the disease, possibly owing to incomplete case coverage. In fact, we also found that the region of Southern Denmark, where the antibodies are tested, had the highest national IR. Given the nature of the centralized antibody testing with complete capture of antibody testing, the regional differences are unlikely to be related to referral bias in Denmark. This points to physician awareness being higher in regions with AE expertise.

Some regional studies have estimated higher IRs than those reported in the national studies. A 2018 study from the Mayo Clinic (Minnesota, United States) found that the IR of AE was comparable to that of infectious encephalitis in Olmsted County (8.0 vs 10.0)[9]. However, the study definition of AE was broad, and the cohort contained patients with AE mediated by antibodies with intracellular and extracellular targets, MOGAD, Bickerstaff encephalitis, Hashimoto encephalopathy, ADEM, autoimmune limbic encephalitis with no antibodies, and probable sero-negative AE.

Comparing the IR of NMDARE, we found that the national IR of NMDARE was similar in Denmark and the Netherlands (Denmark 2019–2023: 1.06 vs. the Netherlands 2016–2021: 0.97). The national NMDARE IR in France was approximately half of this (2016–2018: 0.5), while the IR for the region of the French referral center was 0.9.

Studies reporting regional epidemiology have found NMDARE IRs between 0.3 and 2.3 [9, 14, 31, 33] with higher IRs among some ethnic groups and in certain geographical areas. A previous review and meta-analysis found that the IR of NMDARE was particularly high in southern hemisphere countries far from the equator, compared with Europe and North America [1].

The national IRs of LGI1 were the same in Denmark and France (0.4 vs. 0.4), including the French region with high case-coverage. It was considerably higher in the Netherlands, where NMDARE and LGI1 had similar IRs (0.9). Interestingly, an Italian study found the opposite relationship, with the regional incidence of LGI1 being double that of NMDARE (0.84 vs. 0.42) [33].

Some AE types are associated with specific HLA types [4, 21, 30]. Perhaps, the geographical variation in the incidence of AE and its subtypes is in part a result of differences in genetics. In comparison, the incidence of anti-muscle-specific kinase (MuSK) associated myasthenia, another IgG4 antibody–mediated neurological disease, is characterized by a geographical north–south gradient. In northern Europe, anti-MuSK–mediated disease is exceedingly rare, whereas it is more common the southern Europe, especially in the Mediterranean [7].

Another possibility is that clinician awareness of certain phenotypes is heterogeneous among countries. It is possible that some LGI1 patients in Denmark remain undiagnosed. In fact, a previous study from the Netherlands found that 0,8 percent of patients from two Dutch memory clinics who had received a dementia diagnosis, had antibody positive AE[3]. This is further supported by the already mentioned increased incidence in regions with disease expertise.

In Denmark, we saw that despite the increase in overall antibody testing, the proportion of patients being tested in both serum and CSF, remained largely unchanged at around 30%. To improve the diagnostic utility of autoantibody testing in this disease entity, most patients should have both serum and CSF testing performed simultaneously. This finding could reflect limited physician awareness regarding AE diagnostics and could result in false-negative tests in patients with AE.

The rate of positive tests in Denmark was low (~ 1.4%) which may indicate low pre-test probability of AE among patients tested, although a 2025 study found a similar rate of 1.6% [10].

Taken together, increasing annual testing, stable incidences of NMDARE and LGI1 and new antibodies being discovered continually, the increase in crude IR is likely a result of improved case ascertainment rather than an increase in incident AE. Moreover, considering the geographical variation in incidence, it is probable that some cases of AE are not being diagnosed, especially types of encephalitis with insidious onset that mimic other diseases.

### Treatment

Most AE patients received first-line treatment, but the use of second-line treatment and steroid-sparing agents has changed. In NMDARE there was an increase in the use of second-line treatment. This change in treatment practice could be due to increasing experience with both treating NMDARE patients and using second-line therapies. In fact, a 2025 study found that rituximab treatment was associated with longer time to relapse in NMDARE patients [27], providing evidence for this shift in treatment strategy. The decrease in the use of steroid-sparing agents in treating NMDARE patients could be due to the increase in use of efficacious second-line treatment.

LGI1 patients usually respond very well to first-line treatment, especially corticosteroids. As with NMDARE patients, increasing experience in treating this patient group could have led to a decrease in both second-line and steroid-sparing treatments. A 2023 systematic review and meta-analysis showed that neither the use of second-line treatment nor maintenance immunotherapy was predictors for outcome [22].

### Strengths and limitations

The Danish centralized antibody testing allows for studying the epidemiology of AE in a national, population-based setting, which is a major strength of this study.

Patient data is comprehensive, with detailed clinical, paraclinical, treatment, and outcomes.

Census data from Statistics Denmark are detailed with high validity making regional and age- and sex-adjusted IRs possible to calculate.

A limitation of our study is that data on antibodies with intracellular targets were not included in this study. Further, sero-negative patients were not included either as we currently do not have nationwide coverage of this patient group.

Because patient data are derived from medical records, there are some patients with missing data and functional outcomes scores are estimated.

Some antibodies were discovered during the study period, e.g., GFAP or IgLON5. Hence, some of the increase in overall incidence over time is attributable to new types of AE, rather than increased clinician awareness.

Although the sensitivity of live-cell CBA is superior to that of a fixed CBA, most countries do not have access to live-cell assays and we feel that presenting this real-world data will be of importance to many clinicians.

## Conclusion

In this nationwide, population-based cohort study, we used a unique nationwide, centralized testing setup to identify all sero-positive AE patients diagnosed in Denmark during a 15-year period. We found that Danish AE patients aligned with established literature and that the national IR was comparable to previous findings from European countries. We found that there was an annual 10% increase in the crude IR of AE along with a 23% annual increase in antibody testing. The incidences of NMDARE and LGI1 encephalitis were stable over time, suggesting that the increase in overall incidence is due to improvement in case ascertainment and not an increase in the number of patients. As in a previous epidemiological study from France, we found that regional incidence was highest where the reference center is located, which suggests that not all Danish AE patients are identified.

There was a shift towards second-line treatment over steroid-sparing agents in NMDARE and decreasing use of steroid-sparing agents in NMDARE and LGI1 patients. These trends could be the result of increasing real-world experience with treating this patient group.

Although we now have more than 15 years of collective experience with diagnosing and treating this group of patients, there is a need for improving the detection rate of AE patients. Further knowledge dissemination to relevant institutions could increase physician awareness in places where this highly treatable disease could be mistaken for one of its mimics.

## Supplementary Information

Below is the link to the electronic supplementary material.

Supplementary file1 (DOCX 217 KB)

## Data Availability

The datasets generated and/or analyzed during the current study are not publicly available because they are derived from medical records and the study did not include patient consent for public data sharing. Data use is therefore restricted by the study’s ethical approval and institutional regulations but is available from the corresponding author on reasonable request and with permission from the relevant authorities.
