# Supplementary material for: Fifteen years of autoimmune encephalitis in Denmark: incidence, epidemiology, and trends in treatment
Source: J Neurol. 2026 Jul 23;273(8):488. doi: 10.1007/s00415-026-13979-8 (PMC13395891; doi:10.1007/s00415-026-13979-8)
Supplement: Supplementary file 1 — Supplementary file1 (DOCX 217 KB) [file 415_2026_13979_MOESM1_ESM.docx]

# Supplementary material

## eFigures

### eFigure 1 Antibody distribution, positivity and alternative diagnoses among excluded patients


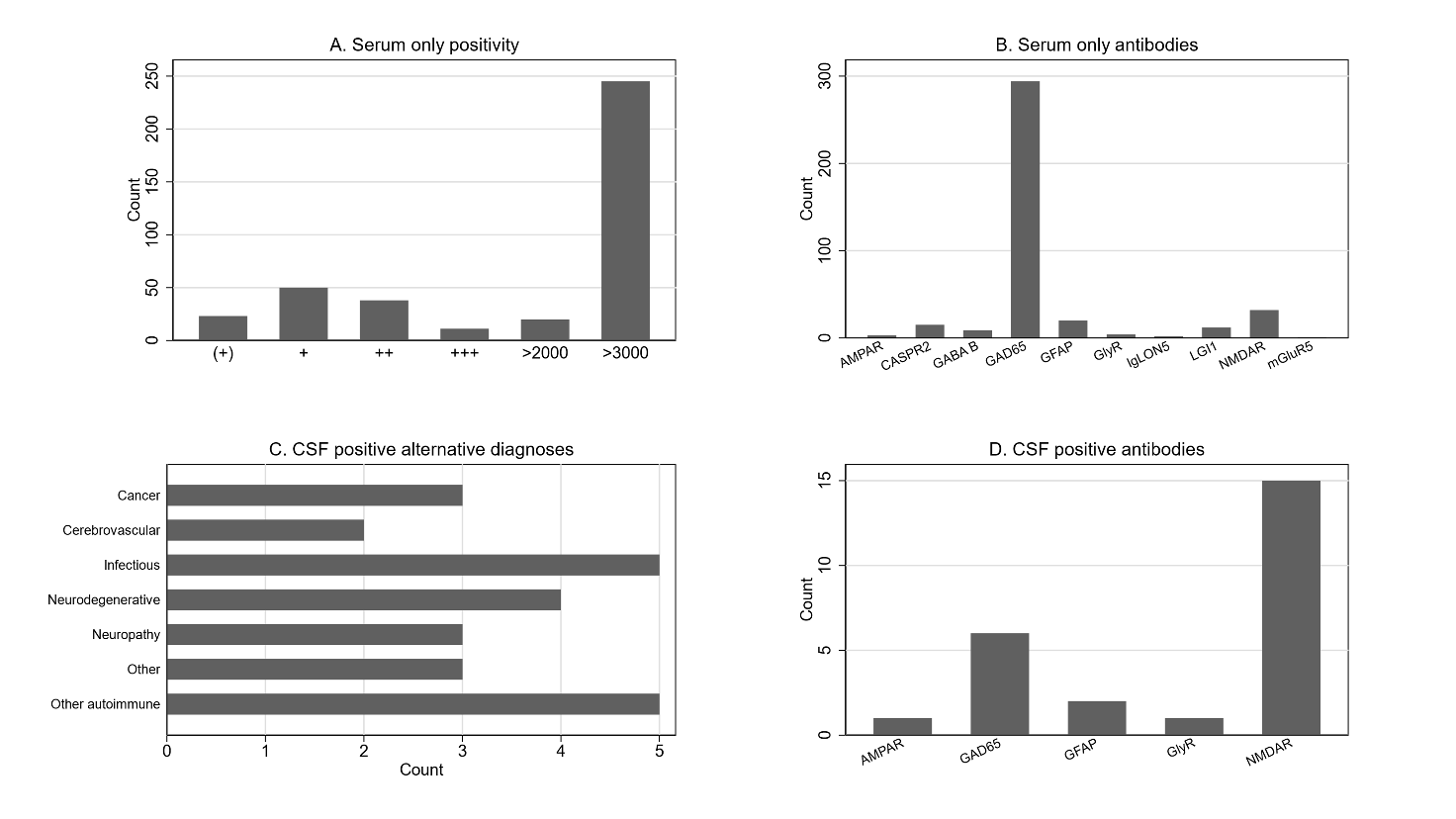


(A) Positivity among excluded patients where antibodies were detected in serum only. Positivity of cell-based assay or LIA (strength of fluorescence from weakest (+) to strongest +++), or positive radioimmunoassay (>2000 IU or >3000 IU). (B) The distribution of antibodies among patients where an antibody was detected in serum only. (C) Alternative diagnoses among patients where an antibody was detected in CSF with or without serum positivity. (D) The distribution of antibodies among those with CSF positivity.

One patient with neurosyphilis figuring here as AMPAR positive in CSF was also positive for LGI1, CASPR2 and GABAB in CSF. Another patient with Lyme disease had strong NMDAR positivity in CSF (previously published as case report[1]).

# References

1. Knudtzen FC, Nilsson AC, Skarphedinsson S, Blaabjerg M (2020) False-positive anti-NMDA receptor antibodies in severe case of Lyme neuroborreliosis. Neurol Sci 41:197-199
